# Perspectives on oral health care integration in HIV care and research in low-resource settings: Insights from patients and key stakeholders in Uganda

**DOI:** 10.21203/rs.3.rs-7925254/v1

**Published:** 2025-11-25

**Authors:** William Buwembo, Laban Muteebwa, Ian G. Munabi, Adriane Kamulegeya, Arabat Kasangaki, Aloysius G. Mubuuke, Katumba Sentongo, Catherine Lutalo Mwesigwa, Annet Kutesa, Patton L. Lauren, Ronald P. Strauss, Fred Collins Semitala

**Affiliations:** Makerere University; Makerere University; Makerere University; Makerere University; Uganda Christian University; Makerere University; Former Registrar Uganda Medical and Dental Practitioners’ Council; Makerere University; Makerere University; University of North Carolina; University of North Carolina; Makerere University

## Abstract

**Background:**

Oral health conditions are common among people living with HIV, yet many have unmet needs for oral health care. In most low-income countries like Uganda, oral health is not yet integrated in the HIV care settings. We aimed to explore the stakeholder’s perspectives on oral health care integration in HIV care and research settings.

**Methods:**

We conducted focused group discussions (FGDs) among people living with HIV (PLHIV) and semi-structured interviews among other key stakeholders that included policy makers, educators and practitioners in fields of HIV and oral health. The participants for both interviews and FGDs were selected purposively. The interviews and FGDs were audio-recorded, transcribed verbatim and analyzed using inductive thematic analysis approach in OpenCode software version 4.03.

**Results:**

Four FGDs were conducted among 31 PLHIV, and interviews were conducted among 18 key stakeholders. The PLHIV acknowledged that oral manifestations are common and were associated with physical, psychological and social consequences which greatly affect their quality of life. They also reported facing stigma and discrimination in non-HIV settings where they seek oral care and advocated for the integration of oral health services in HIV care settings. The barriers to integration of oral health in HIV care identified by the key stakeholders included limited local research to inform policy, lack of clear policy on integration of oral health, inadequate trained health workers and limited resources to support research and integration of oral health services.

**Conclusion:**

The findings highlight the urgent need to integrate oral health services into HIV care settings in Uganda. Addressing policy gaps, stigma, inadequate training, and limited research through stakeholder collaboration and capacity building could enhance access to oral care and strengthen the overall quality of life for people living with HIV.

## Background

Oral health is an essential but often neglected component of comprehensive HIV care, despite its significant impact on the quality of life of people living with HIV (PLHIV). In resource-limited settings like Uganda, oral manifestations have traditionally been used as early indicators and predictors of progression of HIV infection(1, 2). In Uganda, close to 1.4 Million people live with HIV of which about 94% are on life-long antiretroviral therapy (ART) and 94% of them are virally suppressed(3). The robust HIV diagnosis, and medical management has ensured that PLHIV live longer and healthier lives, to almost the same lifespan as non-HIV infected individuals (4). Nonetheless, other studies have reported altered microbiome of the oral cavity in PLHIV (5–7) which negatively impacts their oral health. Oral comorbidities of HIV are associated with a reduced quality of life among PLHIV(8, 9), which have an overbearing effect on HIV outcomes including adherence to ART and nutrition. Recent studies conducted in Uganda reported a disproportionately high burden of oral comorbidities among PLHIV. For example, in a large cohort of adult PLHIV in Uganda, over 66% had undiagnosed periodontal disease (10). Furthermore, a recent study conducted among 748 PLHIV in Uganda, found that about 8 in 10 had dental caries(11).

Despite this high burden of oral health conditions among PLHIV, oral health services are not yet integrated into in HIV care settings and policies in Uganda. This means that PLHIV have to source oral health services from clinics outside the HIV clinics which limits access to and utility of these services leading to delays in diagnosis and treatment. Additionally, various studies in both high income(11) and low-middle income countries(12, 13) have reported that PLHIV often face stigma while accessing oral health services outside of HIV clinics, and this was also acknowledged at the 8th World Workshop on Oral Health and HIV/AIDS (WW8)(14). While the Bali Declaration (2019) on Oral Health in HIV/AIDS (at the WW8) emphasized that oral health practitioners be integral to the HIV/AIDS response(15), these specialists are largely not involved in HIV care settings in Uganda. Furthermore, the role of increased oral health research in PLHIV needed to inform national health policies was highlighted by Bali Declaration (15), and a research agenda was proposed (16). However, in low-middle income countries, HIV-related oral health research is still under-represented and this greatly affects care, undermines universal health coverage and limits local evidence-based policy-making. The integration of oral health into HIV care and research requires a multi-sectoral approach involving policymakers, researchers, educators, healthcare providers and PLHIV. We aimed to explore the perspectives of key stakeholders, including policymakers, HIV and oral health researchers, educators, and PLHIV, on the integration of oral health care and research into HIV care-settings in Uganda.

## Methods

### Study setting and design

The study utilized an exploratory descriptive qualitative study design. The study was conducted in Uganda, an East African country with a population of about 45 Million people and an HIV prevalence of 5.1%(17). In Uganda, about 94% of PLHIV are on ART accessible freely from HIV clinics(3). For this study PLHIV were recruited from high-volume HIV clinics including the Makerere University Joint AIDS Program (MJAP), Mulago ISS clinic and Kisenyi Health Center IV (KHCIV). MJAP is one of the largest non-governmental organizations (NGOs) that gives comprehensive HIV care to about 16,500 PLHIV at Mulago ISS clinic alone with some support from the government of Uganda. KHCIV is a level 5 public/government health facility that offers general medical and surgical services including maternity services, and serves about 1,200 people daily. The HIV care services at KHCIV are free for all PLHIV.

### Study population and eligibility criteria

We enrolled 31 PLHIV in four focused group discussions (FDGs) of which two were conducted at each of the HIV clinics (MJAP and KHCIV). The participants for FGDs were eligible if they had been in HIV care for at least one year and gave written informed consent to participate in the study. We also interviewed 18 key stakeholders including educators, oral health and HIV researchers, policy makers and practitioners from both oral health and HIV care settings. The stakeholders were eligible if they had been in their field for at least five years

### Data collection tools and procedures

The Key Informant Interview (KII) and FDG guides (supplementary file 1) were used to guide the interviews with PLHIV and key stakeholders, respectively. The PLHIV were selected by purposive sampling with the help of their peer-leaders and the FGDs were conducted at the health facilities. All the FGDs were conducted in Luganda, a commonly used local language among the PLHIV that access the selected HIV clinics, and discussions were audio-recorded. The participants for KII were selected purposively and the interviews were conducted in the English language and audio recorded. The participants were either interviewed face to face or online through Zoom after obtaining informed consent. The interviews were conducted by three trained research assistants experienced in qualitative research data collection.

### Data management and analysis

The audio records were transcribed verbatim immediately after the interviews and were backed up on a password-protected external hard drive. The Luganda transcripts were translated into the English language by an experienced translator. The English transcripts were then uploaded in OpenCode software version 4.03 for data analysis. The data analysis followed an inductive thematic analysis approach. The coding was done by two independent analysists who had sufficient engagement with the datasets and documented their preconceived experiences before data analysis. The conflicts in coding were resolved by discussions to generate consensus. Similar codes were summarized into sub-themes, and the sub-themes were summarized into themes. Member checking with PLHIV and stakeholders was done to ensure credibility and accuracy of the findings.

## Results

### Characteristics of FGD participants

Four (4) FGDs were conducted which included 31 participants with a median age of 44.0 years (IQR: 33.0, 50.0). About half were males (16/31), and a similar number (16/31) were from Makerere University Joint AIDS Program (MJAP). The median (IQR) period since first diagnosis of HIV was 12.0 years (IQR: 9.0,19.0). The participants had a median duration on ART of 10.0 years (IQR: 8.0, 16.0) (Table 1).

### Characteristics of stakeholders who participated in key informant interviews

A total of 18 stakeholders participated in KIIs of which 10/18 were males and 14/18 were affiliated to public (government) institutions. About a third were educators in universities and 4/18 were practitioners in oral health or HIV care (Table 2).

### Findings from FGDs conducted among PLHIV

#### Theme 1: Oral conditions among PLHIV

##### Sub-theme 1: Knowledge about common oral conditions

The participants acknowledged that oral conditions were common among PLHIV. They were able to describe some of the common oral condition they have suffered or seen in other PLHIV that included mouth ulcers, painful jaws, whitish thrush, bad breath, dry lips, red lips and fissured gums. For example, a 40-year-old male who has lived with HIV for 18 years recounted that *“Personally, I frequently suffer from pimple like-warts beneath my tongue and outside my mouth. Additionally, I have also suffered from mouth ulcers”*.

Another 38-year-old female who has lived with HIV for 9 years said that *“The other person I interfaced with had pus like-mouth sores and throat sores (mouth ulcers) and she always had difficulties in eating or drinking hot foodstuffs”*.

##### Sub-theme 2: Perceived causes and triggers

The PLHIV thought that the oral health conditions are due to poor hygiene and dehydration associated with taking the ART pills.

A 44-year-old male who has lived with HIV for 11 years reported that *“Dehydration is the cause of oral health diseases among PLHIV because ART pills are strong pills, and unfortunately, most clients swallow them with less water consumption.”*

#### Theme 2: Impact of oral issues on activities of daily living

##### Sub-theme 1: Physical/Nutrition issues

The PLHIV highlighted the challenges posed by untreated oral comorbidities in performing their physical functions including eating. They reported that presence of oral conditions affects their feeding habits including loss of appetite and failure to eat cold or hot foods, but also have challenges in taking their ART pills.

“First of all, the oral health diseases (OHD) expose PLHIV to eating discomforts and constraints (failure to eat hot foodstuffs) especially the mouth sores (mouth ulcers)”

–40-year-old male who has lived with HIV for 18 years

“Oral health diseases such as mouth ulcers can lead to limited chances of eating foodstuffs of our choice like then corns and maize.”

– 50-year-old female who has lived with HIV for 16 years

##### Sub-theme 2: Psychological and social impact

The PLHIV elaborated that oral manifestations make them feel uncomfortable in their social groups. This is because they feel that they may be stigmatized (perceived stigma) since the oral manifestations have traditionally been linked to compromised immunity secondary to HIV/AIDS.

“Oral health diseases among PLHIV subject them to stigma and low esteem. I sometimes wonder whether the people in the society have gotten to know.”

- 40-year-old male who has lived with HIV for 18 years

“Honestly speaking, whenever I’m disturbed by the red lips or any other related-health complications, it gets hard for me to freely move around my own community because of the fear of being segregated and subjected to stigma.”

– 34-year-old female who has lived with HIV for 4 years

#### Theme 3: Challenges in accessing oral health services

##### Sub-theme 1: Financial/logistical barriers

PLHIV often receive comprehensive medical care at most of the HIV clinics free of charge, however they expressed concerns that despite oral conditions being common, oral health isn’t integrated in the HIV care. Therefore, PLHIV have to incur the cost of oral care by themselves outside of the HIV clinics including buying the medicines for oral conditions. The lack of sufficient funds impedes access to oral care for PLHIV, yet oral-health related challenges greatly affect HIV outcomes such as adherence to the ART pills

“Most of the PLHIV are financially unstable to access the oral health services. For example, there’s a time I failed to purchase the treatment of mouth ulcers of 180k because I was economically unstable and yet it was feeling a lot of pain.”

– 52-year-old male who has lived with HIV for 13 years

“Lack of enough funds to access oral health medical services by the ART patients is one of the limiting factors.”

– 50-year-old female who has lived with HIV for14 years

##### Sub-theme 2: Stigma and fear of disclosure

PLHIV also displayed their discomfort in being treated by dental practitioners outside the HIV clinics because often don’t want to disclose their HIV status. Despite HIV status and drug history being important in guiding clinical care of their oral conditions, PLHIV fear being stigmatized when they seek care in non-HIV-specific clinics. Moreover, PLHIV reported they lack knowledge about oral health and sometimes long endure untreated oral conditions which affect their quality of life.

“Fear of revealing our HIV status to the strange clinicians in the local clinics is one of the limiting factors for easy access to the oral health services as PLHIV. In most cases before the local clinicians prescribe any drugs to us, they often ask if we have any other diseases that we are treating that might have caused the oral health diseases, a question that’s hard to respond to and yet it’s key to be answered in the medical field.”

– 44-year-old male who has lived with HIV for 11 years

“Stigma which is associated to fear of freely opening up about my HIV status to the local clinic owners in my place of residence is one of the limitation factors to the access of medical services for the treatment of oral health diseases.”

– 38-year-old female who has lived with HIV for 9 years

#### Theme 4: Recommendations for improving oral health among PLHIV

##### Sub-theme 1: Integration of oral health in HIV care

The PLHIV felt that oral health services should be integrated in the general HIV services so that they can easily access these services during their routine visits at the ART clinic/centers. However, the participants felt PLHIV should be better educated/informed, so that they are aware of the oral conditions but also seek specialized support to accurately diagnose and treat them. The PLHIV further recommended training of the current HIV care givers so that they can handle their oral health needs.

“I suggest the oral health services should be delivered directly to PLHIV from the ART clinics other than other Entry points or clinics because of the fear and stigma inflicted on us by some health workers.”

– 46-year-old female who has lived with HIV for 19 years

“Provision of medical services of oral health diseases among PLHIV should rather be stationed at the ART clinics other than the village level so as to curb stigma issues that might rise from the local people.”

– 50-year-old female who has lived with HIV for 16 years

##### Sub-theme 2: Stakeholder involvement and engagement

The PLHIV felt that adequate stakeholder engagement is important to address the issues related to oral health. Involving key stakeholders like the Ministry of Health, their peer-leaders, community leaders, community-based organizations and other civil society organizations can go a long way in supporting integration of oral health services in HIV care.

“The Ministry of Health should spearhead the prevention program of oral health diseases among PLHIV to make it easier for other organizations to give a hand.”

– 55-year-old male who has lived with HIV for 10 years

### Findings from the key informant interviews

#### Theme 1: Limited integration of oral health in HIV care

##### Sub-theme 1: Neglected role of oral health

The participants felt that much as oral health is an important aspect that impacts the quality of care of PLHIV, it is often ignored and not prioritized at the ART clinics. They also acknowledge that PLHIV often refrain from discussing oral issues openly with care providers, knowing that these issues are neither prioritized nor addressed in ART clinics. The neglect of oral health issues impacts HIV outcomes and also quality of life.

“Oral health hasn’t been given attention, even though it clearly affects the quality of life.”

- (KII 004, policy maker)

“ART clinics ignore oral health issues even though patients report them.”

- (KII 003, HIV practitioner)

##### Sub-theme 2: Limited research and absence of policies on Integration of oral health in HIV care

The participants felt that integration of oral health into HIV care is hindered by the absence of formalized policies and insufficient research to guide implementation. There are no established guidelines or routine screening protocols for oral health in ART clinics, and minimal studies exist to highlight the burden of oral health conditions among PLHIV. This lack of evidence limits advocacy efforts, leaving policymakers and stakeholders without the data needed to justify and prioritize integration

“Integration [of oral health and HIV] is the way forward, but we lack data to inform decision-making.”

- (KII 002, Policy maker)

“Without evidence, integrating oral health into HIV care remains a distant goal.”

- (KII 004, Policy maker)

“There’s no evidence to show how prevalent oral health issues are among PLHIV.”

- (KII 005, HIV practitioner)

#### Theme 2: Barriers to oral health integration in HIV care

##### Sub-theme 1: Limited resources and expertise

The participants emphasized that the shortage of funding and skilled dental professionals dedicated to this area further constrains research efforts, perpetuating a gap in both care and evidence needed to drive integration of oral healthcare into HIV programs. Moreover, there is limited resources and expertise for oral health research that pose significant challenges to addressing oral health among PLHIV. ART clinics lack essential infrastructure, such as dental equipment and facilities, and have insufficiently trained staff to identify and manage oral health conditions

“Few dentists prioritize this area, and ART clinic health workers lack the expertise to screen for oral health diseases.”

(KII 001, Policy maker)

“We need funding for oral health research and to train clinicians to manage these conditions.”

(KII 005, HIV practitioner)

##### Sub-theme 2: Patient disclosures challenges

HIV practitioners mentioned that many patients fear discussing oral health problems because it is often perceived as indicators of advanced HIV. This reluctance is exacerbated by their awareness that oral health issues are not prioritized in ART clinics, leaving them hesitant to seek help or report symptoms during consultations. Such challenges with patient disclosures significantly hinder the identification and management of oral health issues among PLHIV.

“Patients fear to discuss oral health issues because they’re worried it signals advanced HIV. People hide their oral health problems, knowing ART clinics don’t address them.”

- (KII 003, HIV practitioner)

“Even when we ask, they don’t open up, knowing we lack the resources to help.”

- (KII 005, HIV practitioner)

#### Theme 3: Need for capacity building and evidence generation

##### Sub-theme 1: Training health workers in integrated HIV/oral health care and research

Health workers need practical skills and tools to conduct oral health research, and to identify, screen, and manage oral health conditions among PLHIV. Emphasis should be placed on long-term mentorship programs like masters, fellowship and Doctor of Philosophy (PhD) to ensure hands-on learning and sustained capacity building. However, participants also highlighted the importance of short-term trainings for non-specialized clinicians, nurses and other healthcare providers who work in HIV clinics to support HIV-oral health care and research. Stakeholders emphasize the need for a multidisciplinary approach to mentorship to support the mentees to hone their knowledge and skills in both oral health and HIV care and research.

“Let the training be all encompassing. let training not only consider people in oral health say dentists or HIV but open it up to people with potential and interest to grow their career in [HIV/oral health] care and research”

- (KII 08, Associate Professor)

“Let us propose inclusion of specific oral health sessions in the training curriculum of the health workers in the different fields because oral health is in every area only that it’s not yet realized”

- (KII 18, Associate Professor)

##### Sub-theme 2: Local evidence generation to support policy

The participants consistently recounted the importance of generating local evidence on the burden and impact of oral health issues among PLHIV to inform policies, attract funding, and justify integrating oral health into HIV care.

“Once we show oral health impacts ART adherence, we’ll get their [policy makers] attention.”

(KII 004, policy maker)

“Without local evidence, no one will fund or prioritize oral health.”

- (KII 006, oral health practitioner)

“Evidence will drive policymakers to support integration.”

- (KII 005, HIV practitioner)

#### Theme 4: Collaborative approaches to integrating oral health in HIV care

##### Sub-theme 1: Multidisciplinary Collaboration

The participants emphasized that fostering teamwork across disciplines ensures comprehensive care for patients, reduces fragmentation in services (working in silos), and promotes the alignment of research and clinical efforts to address the oral health needs of PLHIV effectively. The cooperation between dentists, HIV clinicians, policy makers and other stakeholders in developing oral health care and research priorities will ensure broadened available evidence to inform holistic care of PLHIV. Through such multidisciplinary-informed research, oral health screening tools and care protocols can be developed and integrated into the HIV care.

“Dentists and HIV clinicians should co-develop tools for oral health screening.”

- (KII 003, HIV practitioner)

“HIV patients deserve holistic care from all [HIV and oral health] specialists.”

- (KII 004, Policy maker)

“Collaboration between disciplines is the future of integrated care.”

- (KII 005, HIV practitioner)

##### Sub-theme 2: Leveraging existing infrastructure

Participants recounted that some existing platforms like the ART clinics provide an opportunity to access patients for oral health related research but also provide oral care. The PLHIV often visit the ART clinics for ART refills and other comorbidity management and thus provide an opportunity to screen, manage and conduct research. Furthermore, the participants highlighted that the existing training programs at Makerere University provide an opportunity for cross-learning and multidisciplinary collaborations to support HIV/oral health research training.

“We can start by screening oral health during ART visits and referring severe cases.”

- (KII 002, Policy maker)

“We already have ART clinics, so adding oral health screening is feasible.”

- (KII 005, HIV practitioner)

“Referral systems should ensure patients get the oral care they need.”

- (KII 003, HIV practitioner)

## Discussion

### Oral health burden, impact and access for PLHIV

The study findings revealed that oral health conditions are common among PLHIV yet they remain largely unaddressed in HIV care settings. Indeed, other Ugandan studies have reported a high prevalence of oral conditions in PLHIV. For example, a survey among a large cohort of adult PLHIV reported that over 66% of PLHIV had periodontal disease (10). A another recent study reported that about 8 in 10 PLHIV had dental caries, of which sex and duration on ART had significant influence on development of dental caries (11). Furthermore, a recent meta-analysis reported a higher risk of dental caries among children and adolescents living with HIV compared to those not living with HIV (18). In our current study, PLHIV reported feeling unease with visiting dental clinics which are outside the HIV clinic setting, and most of them felt that they would not disclose their HIV status to the oral health practitioners. Moreover, PLHIV in this study reported significant psychosocial distress due to oral health complications, with some individuals experiencing low self-esteem and avoiding social interactions. Several reports have also highlighted that PLHIV are stigmatized in non-HIV settings like oral health clinics (12–14, 19) which range from discriminatory to refusal of care. For example, in a study done in the United Kingdom (UK), about 2 in 10 PLHIV reported being treated differently from other patients attending the dental practice (19). Nonetheless, such discrimination often happens more frequently in low-income countries like Uganda compared to high-income countries like the UK, consequently affecting utilization of oral health services. Recent literature has similarly reported chronic low uptake/utilization of dental services among PLHIV (20, 21), which undermines universal health coverage. This not only leads to late diagnosis and treatment, reduced quality of life, uncertain treatment outcomes (due to potential drug interactions with antiretroviral drugs), but also affects adherence to ART which threatens patient outcomes like viral suppression. Integration of oral health services in HIV care settings which have less stigmatization could help in improving access and utilization of these services. The sub-optimal utilization of oral health services greatly affects the oral health-related quality of life because PLHIV stay with untreated oral conditions for longer time periods. For example, in this study, PLHIV revealed that oral health conditions negatively impact daily activities, including nutrition and medication adherence. Similarly other studies have reported a very low oral health-related quality of life among PLHIV (8, 22–24). For example, a recent scoping review of studies done in South Africa reported that dysphagia (difficulty in swallowing) secondary to oral health conditions among adult PLHIV affected pill swallowing and influenced adherence to ART (25).

### Oral health integration in HIV care settings

The stakeholders including PLHIV underscored the need for integrating oral health services into HIV care settings to optimize uptake which will ensure early diagnosis, treatment and improved quality of life among PLHIV. This integration has to happen from policy level to clinical care settings where oral health practitioners work with other health professionals that interact with PLHIV. In Uganda, the integration of other services like screening and management of non-communicable diseases (NCDs) like hypertension, mental disorders and diabetes have been successful and included in national HIV management guidelines (26), yet oral health remains neglected. The stakeholders highlighted the barriers to integration of oral health into HIV care including lack of research evidence, health professional working in silos, inadequate trained staff and policy gaps. A recent scoping review documented similar challenges impeding the integration of dental services in primary care settings (27).

The stakeholders emphasized the need for more research to support local evidence synthesis so that policy makers can utilize such evidence to support integrated oral health services in HIV care and policies. Nevertheless, the stakeholders highlighted the need to empower health professionals both from HIV care and oral health disciplines to effectively collaborate to address the oral health needs of PLHIV. Such empowerment can be achieved through a target capacity building program for not only clinical staff, but also researchers to close the oral health care and knowledge gaps and help to generate local evidence to support policy changes.

## Conclusions

This study highlights the urgent need to integrate oral health services into HIV care settings in Uganda, guided by the voices of PLHIV and key stakeholders. Addressing policy and research gaps, stigma, and the limited training of healthcare workers through multi-sectoral collaboration is critical. Strengthening capacity for HIV/Oral health research and embedding oral health screening and treatment within ART clinics could enhance service uptake, reduce stigma, and improve ART adherence and quality of life. The findings provide actionable evidence to inform Uganda’s Ministry of Health and academic institutions in developing integrated HIV/Oral health care and research training frameworks

## Supplementary Material

Supplementary Files

This is a list of supplementary files associated with this preprint. Click to download.

- FGDKIIguides.pdf

## Figures and Tables

**Table 1:** The demographic characteristics of 31 participants of FGDs conducted at Kisenyi HCIV and MJAP HIV clinics

Variable	Categories	Frequency (N = 31)	Percentage (%)
Age (years)	Median (IQR*)	42.0 (33.0, 49.0)	
Sex	Male	16	53.3
	Female	15	46.7
Health facility	Kisenyi HCIV**	15	46.7
	MJAP***	16	53.3
Period since diagnosis (years)	Median (IQR)	12.0 (9.0, 19.0)	
Period since initiation of ART (years)	Median (IQR)	10.0 (8.0, 16.0)	

* IQR-Interquartile range

** MJAP-Makerere University Joint AIDS Program,

*** HCIV-Health Centre IV

**Table 2:** Demographic characteristics of 18 stakeholders who participated in Key Informant Interviews

Variable	Categories	Frequency (N = 18)	Percentage (%)
Sex	Male	10	55.6
	Female	8	44.4
Institutional affiliation	Public	14	77.8
	Private/NGO*	4	22.2
Stakeholder group	Policy maker	4	22.2
	Educator	6	33.4
	Researcher	4	22.2
	Practitioner	4	22.2

* NGO-Non-Governmental Organization

## Data Availability

The datasets used during the analysis for this qualitative inquiry are available from the corresponding author upon reasonable request.
